# Risk factors for second primary esophageal cancer after head and neck squamous cell carcinoma: a systematic review and meta-analysis

**DOI:** 10.1016/j.esmogo.2026.100351

**Published:** 2026-06-30

**Authors:** T. De Bock, A.D.I. Maan, R.H.A. Verhoeven, S. Van Roy, O. Lenssen, D. De Cock, A.D. Koch, M.T. Brands

**Affiliations:** 1 Department of Oral and Maxillo-Facial Surgery, UZ Brussel, Brussels, Belgium; 2 Department of Surgery, Faculty of Medicine and Pharmacy, Vrije Universiteit Brussel (VUB), Brussels, Belgium; 3 Department of Gastroenterology and Hepatology, Erasmus MC Cancer Institute, University Medical Center Rotterdam, Rotterdam, the Netherlands; 4 Department of Research & Development, Netherlands Comprehensive Cancer Organization (IKNL), Utrecht, the Netherlands; 5 Department of Medical Oncology, Amsterdam UMC location University of Amsterdam, Amsterdam, the Netherlands; 6 Department of Cancer Treatment and Quality of Life, Cancer Center Amsterdam, Amsterdam, the Netherlands; 7 Department of Oral and Maxillo-Facial Surgery, ZAS Middelheim, Antwerp, Belgium; 8 Biostatistics and Medical Informatics Research Group, Research Centre for Digital Medicine (REDM), Department of Public Health, Faculty of Medicine and Pharmacy, Vrije Universiteit Brussel (VUB), Brussels, Belgium

**Keywords:** esophageal carcinoma, head and neck squamous cell carcinoma, risk factors, screening

## Abstract

**Background:**

Patients with head and neck squamous cell carcinoma (HNSCC) are at an elevated risk of developing second primary tumors. Esophageal second primary tumors (ESPTs) represent a significant clinical challenge due to their frequent late detection and associated poor prognosis. This systematic literature review aims to identify and synthesize current evidence on risk factors for synchronous and metachronous ESPTs in patients with HNSCC. This can help identify individuals at highest risk so that targeted screening strategies can be created, which could ultimately improve long-term outcomes through earlier diagnosis.

**Materials and methods:**

A comprehensive search was conducted in PubMed, Web of Science, and Embase using a predefined search strategy. Eligible studies included observational, cohort, and case-control analyses involving adult patients with histologically confirmed HNSCC, either with or without an ESPT. Studies focusing exclusively on genome-associated risk factors, or those not addressing risk factors for ESPTs, were excluded. Meta-analyses were carried out when at least five studies reported on a specific risk factor.

**Results:**

Of 1338 records screened, 22 studies met the inclusion criteria and were included. In the meta-analyses, the following risk factors were found to be statistically significant for the development of ESPTs among HNSCC survivors: male sex [odds ratio (OR) 4.73; 95% confidence interval (CI) 2.15-10.40; *P* = 0.0001], HNSCC location in the hypopharynx (OR 4.31; 95% CI 2.13-8.75; *P* < 0.0001) or oropharynx (OR 2.40; 95% CI 1.80-3.20; *P* < 0.0001), alcohol consumption (OR 5.45; 95% CI 2.29-13.38; *P* = 0.0001), and stage IV disease of the first primary HNSCC (OR 1.90; 95% CI 1.16-3.12; *P* = 0.0110).

**Conclusions:**

Male sex, HNSCCs located in the hypopharynx or oropharynx, advanced tumor stage, and heavy alcohol consumption are associated with an increased risk of ESPT development. These findings may help identify susceptible patient subgroups who could benefit from future risk-adapted esophageal surveillance strategies.

## Highlights

• Head and neck cancer survivors have an increased risk of second primary esophageal tumors.
• Esophageal cancer after head and neck cancer has a bad prognosis.
• Identification of risk factors is associated with a higher risk of second primary esophageal cancer after head and neck cancer.
• A high-risk group may benefit from screening.

## Introduction

The Global Cancer Statistics report of 2022 identified head and neck squamous cell carcinoma (HNSCC) as the sixth most common malignancy, underscoring the substantial global health burden associated with HNSCC.^1^ Consequently, the management of long-term effects associated with HNSCC, such as the elevated risk of developing a second primary tumor (SPT), should be addressed.^2^^,^^3^

Individuals with a history of a primary malignancy of the head and neck are at an elevated risk of developing SPTs of the esophagus (ESPTs), often explained by the shared carcinogen exposure such as smoking or alcohol consumption.^2^ ESPTs pose a significant clinical challenge due to their often late detection and associated poor prognosis.^4^ Therefore, early detection through screening might be recommended for certain patient populations as proposed by a recent systematic review on the yield of screening for ESPTs.^5^

The incidence of ESPTs in patients with HNSCC is relatively low (2%-5%), making routine screening of the entire HNSCC population likely not justified.^6^, ^7^, ^8^ As the understanding of SPTs continues to evolve, there is an increasing consensus in literature advocating for personalized follow-up strategies for HNSCC patients.^9^^,^^10^ However, current clinical guidelines recommend only a standardized follow-up approach for all HNSCC patients focusing on recurring and metastatic HNSCC. It involves a clinical visit every 2-3 months for the first 2 years, every 6 months from years 3 to 5, and annually thereafter.^11^ Imaging is reserved for symptomatic patients or those with clinical symptoms. However, this approach does not screen for ESPTs, which are often detected late as symptoms typically arise only once the disease is advanced. To optimize the clinical utility and cost-effectiveness of prospective screening programs, further stratification based on individualized risk profiles should be implemented. In order to create these risk profiles, this systematic review aims to identify current evidence on risk factors for ESPTs in patients with a history of HNSCC.

## Materials and Methods

### Literature search and selection criteria

A systematic literature search was conducted in *Web* *of Science,* *PubMed,* and *Embase* on 18 March 2025, with all sources last updated on 1 September 2025. The complete search strategies for each database are provided in the Supplementary Data Forms S1-S3, available at https://doi.org/10.1016/j.esmogo.2026.100351.

Studies were included if they met the predefined inclusion criteria regarding study population, intervention/exposure, and outcome measures. Articles were excluded if the full text was unavailable or if they did not specifically address risk factors for ESPT. Inclusion and exclusion criteria are summarized below. The systematic literature review was registered in the Open Science Framework before initiation.^12^

Inclusion criteria

• Previous diagnosis of HNSCC.
• HNSCC including the oral cavity, oropharynx, hypopharynx, and larynx.
• Risk factors for synchronous or metachronous (>6 months after the index tumor) ESPTs.

Exclusion criteria

• Article written in a language other than English.
• Study type: case report, systematic review.
• No abstract available.
• History of non-squamous cell carcinoma of the head and neck region.
• SPTs of other regions than the esophagus, recurrence, or metastasis of the primary HNSCC.
• Genome-associated risk factors.
• Odds ratios (ORs) were not reported or retrievable due to missing exact numbers of patients with and without ESPTs.

All retrieved records were imported into *Rayyan* for screening.^13^ TB and AM independently screened titles and abstracts in the first stage, followed by full-text screening of potentially eligible studies. Disagreements regarding inclusion or exclusion were resolved through discussion until consensus was reached.

### Data extraction and synthesis

An Excel file was prepared before data extraction, listing all variables of interest. Data were initially extracted using a predefined prompt with the assistance of ChatGPT (Supplementary Data Form S4, available at https://doi.org/10.1016/j.esmogo.2025.100257), after which all extracted information was manually verified by the authors. For each included study, we aimed to convert all reported results into ORs with 95% confidence intervals (CI) and *P*-values for uniformity. *P*-values were determined using Fisher’s exact test. When studies included overlapping patient populations, analyses were carried out using the study with the highest number of inclusions. As synchronous tumors are already actively searched for in the initial staging workup of HNSCC in some countries, we additionally assessed the defining risk factors for metachronous tumors specifically.

The quality of the studies was assessed using the Methodological Index for Non-Randomized Studies (MINORS).^14^ A meta-analysis was carried out when at least five studies described a specific risk factor. All meta-analyses were conducted in R software (version 2023.12.0), applying a random-effects model to estimate the association between each identified risk factor and the development of ESPTs. The *I*^2^ statistic was used to evaluate heterogeneity across studies.

## Results

### Study selection

**Figure 1:**
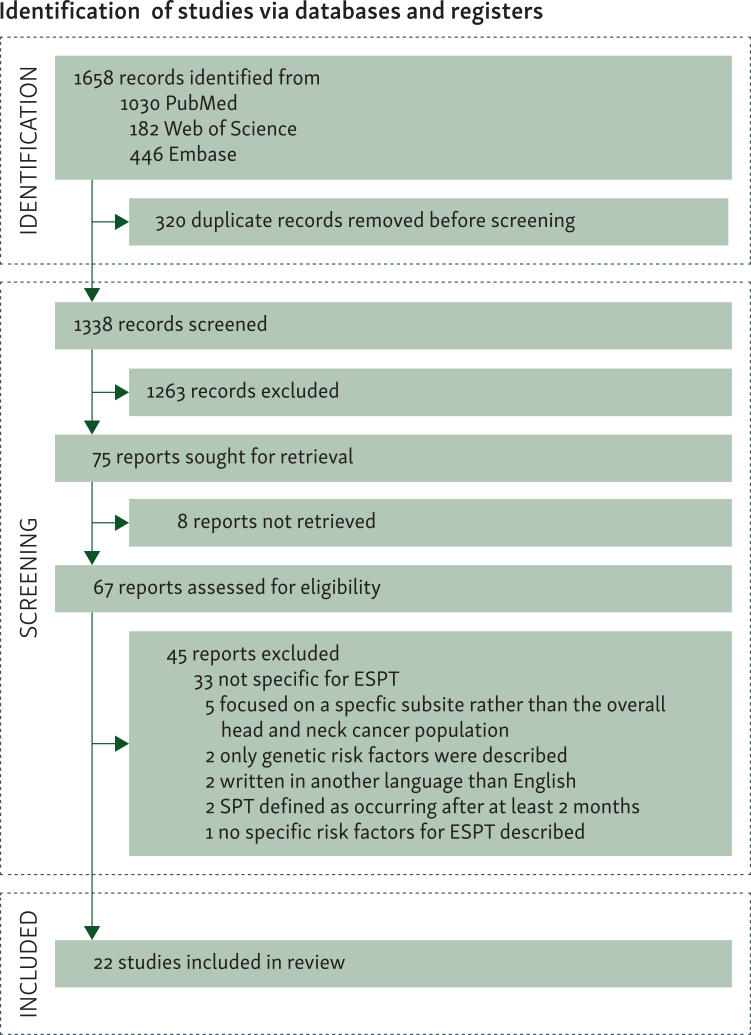
**PRISMA flow diagram of the selection process of articles**. ESPT, esophageal second primary tumor; SPT, second primary tumor.

The study selection process is summarized in the Preferred Reporting Items for Systematic Reviews and Meta-Analyses (PRISMA) flow diagram (Figure 1). The literature search identified 1658 studies. After removal of duplicates and screening of titles and abstracts, 67 studies remained for full-text screening. Any discrepancies between reviewers were discussed until consensus was reached. Ultimately, 22 studies met all inclusion criteria.^6^^,^^15^, ^16^, ^17^, ^18^, ^19^, ^20^, ^21^, ^22^, ^23^, ^24^, ^25^, ^26^, ^27^, ^28^, ^29^, ^30^, ^31^, ^32^, ^33^, ^34^, ^35^

The risk of bias and methodological quality of each study, which were evaluated using the MINORS criteria, did not lead to exclusion of any studies. An overview of the included studies is provided in Table 1, and the MINORS scoring can be found in the Addendum in Supplementary Materials, available at https://doi.org/10.1016/j.esmogo.2025.100257.

**Table 1:** Study characteristics of the 22 studies included

Authors	Year	Country	Design	N	Risk factors assessed
Petit et al.^15^	2001	France	Pro	1560	HNSCC location
Yamamoto et al.^16^	2002	Japan	Retro	1609	Sex, HNSCC location
Muto et al.^17^	2002	Japan	Retro	227	LVLs
Hashibe et al.^18^	2005	USA	Retro	30221	Radiotherapy
Chow et al.^19^	2009	China	Retro	118	Sex, age, HNSCC location, smoking, alcohol
Wang et al.^20^	2011	Taiwan	Pro	315	Sex, age, HNSCC location, smoking, alcohol, betel quid chewing
Hori et al.^21^	2011	Japan	Retro	1060	LVLs
Kim et al.^22^	2014	Taiwan	Pro	308	Sex, age, HNSCC location, smoking, alcohol, piriform sinus involvement
Lim et al.^23^	2015	Korea	Retro	714	Sex, age, HNSCC location, smoking, alcohol
Huang et al.^24^	2016	Taiwan	Pro	248	Age, alcohol
Wang et al.^25^	2016	Taiwan	Pro	145	Age, smoking, alcohol, betel quid chewing, p16-status, BMI, family history SCC
Gong et al.^26^	2016	Korea	Pro	458	HNSCC location, smoking, alcohol, HNSCC stage
Wang et al.^27^	2017	China	Pro	815	Sex, age, HNSCC location, smoking, alcohol, betel quid chewing
Ni et al.^28^	2018	China	Retro	160	Sex, age, HNSCC location, smoking, alcohol, family history SCC
Wang et al.^29^	2019	Taiwan	Pro	586	Age, HNSCC location, smoking, alcohol, betel quid chewing, HNSCC stage
Ho et al.^30^	2020	China	Retro	702	Sex, HNSCC location, smoking, alcohol
Chen et al.^31^	2020	Taiwan	Pro	175	HNSCC location, smoking, alcohol, betel quid chewing, LVLs, HNSCC stage
Thakur et al.^32^	2021	India	Pro	300	Sex, Age, HNSCC location, smoking, alcohol
Brands et al.^6^	2021	Netherlands	Retro	11263	Age, HNSCC location
Milliet et al.^33^	2021	France	Retro	1291	Sex, HNSCC location, smoking, alcohol, p16-status
Zhu et al.^34^	2022	USA	Retro	2771	Radiotherapy
Wang et al.^35^	2024	Taiwan	Retro	717	Sex, age, HNSCC location, smoking, alcohol, betel quid chewing, BMI, HNSCC stage

BMI, body mass index; HNSCC, head and neck squamous cell carcinoma; LVL, Lugol-voiding lesion; N-stage, nodal stage; Pro, prospective; Retro, retrospective; SCC, squamous cell carcinoma; T-stage, tumor stage.

### Study findings

#### Meta-analyses

Meta-analyses were carried out for the following risk factors: sex, HNSCC location, alcohol and smoking habits, and HNSCC stage. The studies by Wang et al.^20^^,^^25^^,^^27^^,^^29^ included overlapping patient populations. Therefore, for each risk factor represented in more than one of these studies, the study with the largest sample size was selected for analysis, in accordance with the criteria described in the ‘Materials and Methods’ section.

Statistically significant associations were observed for male sex (OR 4.73; 95% CI 2.15-10.40; *P* = 0.0001), HNSCC location in the hypopharynx (OR 4.31; 95% CI 2.13-8.75; *P* < 0.0001) or oropharynx (OR 2.40; 95% CI 1.80-3.20; *P* < 0.0001), alcohol consumption (past or current alcohol consumers) (OR 5.45; 95% CI 2.29-13.38; *P* = 0.0001), and stage IV disease of the index tumor (versus stage I) (OR 1.90; 95% CI 1.16-3.12; *P* = 0.0110).

**Figure 2:**
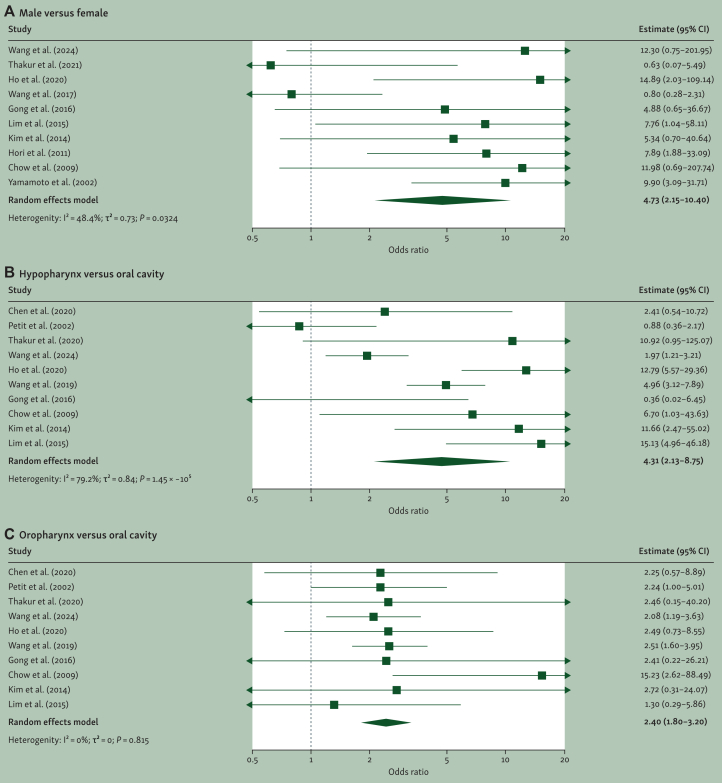

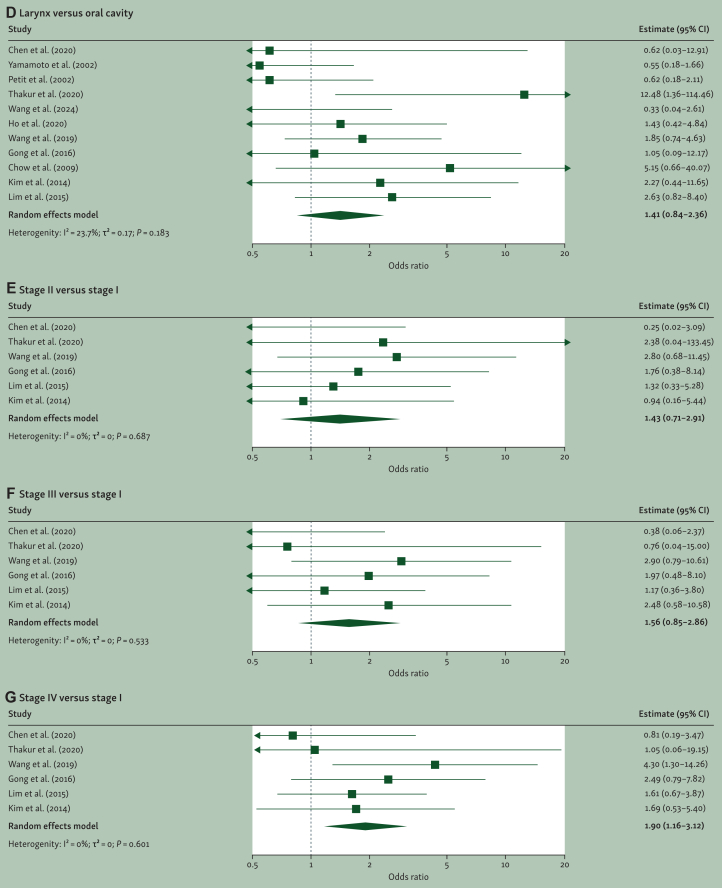

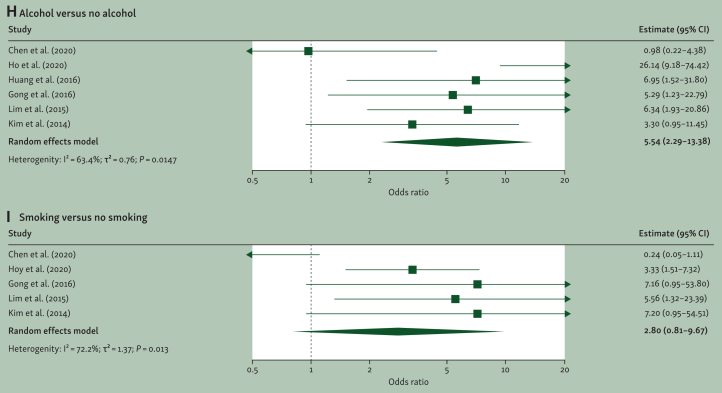
**Forest plots illustrating the odds ratios and corresponding 95% confidence intervals for various risk factors across individual studies**: (A) comparison of male and female patients; (B) comparison of primary tumors in the hypopharynx versus the oral cavity; (C) comparison of primary tumors in the oropharynx versus the oral cavity; (D) comparison of primary tumors in the larynx versus the oral cavity; (E) comparison of stage II versus stage I head and neck cancer; (F) comparison of stage III versus stage I head and neck cancer; (G) comparison of stage IV versus stage I head and neck cancer; (H) comparison of current and past alcohol drinking versus no alcohol consumption; (I) comparison of current and past smoking habits versus no smoking.

No statistically significant associations were found for primary tumor location in the larynx (OR 1.41; 95% CI 0.84-2.36; *P* = 0.1935), stage II disease of the index tumor (OR 1.43; 95% CI 0.71-2.91; *P* = 0.3185) as well as stage III (OR 1.56; 95% CI 0.85-2.86; *P* = 0.1532), and smoking (past or current smokers) (OR 2.80; 95% CI 0.81-9.67; *P* = 0.1024). The corresponding forest plots are presented in Figure 2.

#### Description of additional evaluated risk factors

Due to considerable heterogeneity across study designs, populations, and outcome definitions, as well as the limited number of available studies, quantitative meta-analyses could not be carried out for the additional factors that were evaluated. Findings from individual studies are summarized below.

Lugol-voiding lesions (LVLs) were associated with an increased risk of ESPT, particularly when lesions were of high-grade (OR 5.03; 95% CI 1.52-15.67; *P* = 0.0083) or irregular in shape (OR 12.15; 95% CI 2.49-59.3; *P* = 0.0003).^17^^,^^21^^,^^31^ One study also highlighted the size [≥10 mm; hazard ratio (HR) 5.9, 95% CI 1.3-42.8; *P* = 0.022] and number (≥20; HR 15.7; 95% CI 2.7-129.3; *P* = 0.0021) of LVLs as independent predictors of ESPT occurrence.^17^ All studies were carried out in Asian countries and had a retrospective design including at least 175 patients.

Most findings regarding age suggested that older age was a protective factor for developing ESPTs.^19^^,^^22^, ^23^, ^24^^,^^29^^,^^32^^,^^35^ However, no statistically significant association was reported. All studies were conducted in Asian countries. Four studies were prospective. The minimum total number of patients included was 118.

A negative p16 status was also investigated as a potential risk factor in two studies.^25^^,^^33^ Milliet et al.^33^ reported a significant association (OR 2.08; 95% CI 1.37-3.15; *P* = 0.0004), whereas Wang et al.^25^ found a non-significant trend (OR 1.87; 95% CI 0.51-6.85; *P* = 0.5246). Milliet et al.^33^ conducted a retrospective study in France, whereas Wang et al.^25^ did a prospective study in Taiwan.

Furthermore, radiotherapy following the index HNSCC diagnosis was significantly associated with the development of ESPTs in both studies reporting this risk factor.^18^^,^^34^ Zhu et al.^34^ showed an OR of 3.03 (95% CI 1.12-8.20; *P* = 0.02) among patients receiving postoperative beam radiation therapy for young-onset head and neck cancer. Similarly, Hashibe et al.^18^ found an OR of 3.40 (95% CI 1.24-9.34; *P* = 0.02) among patients treated with radiotherapy, including beam radiation, radioactive implants and/or radioisotopes, for oral cavity HNSCC. Both studies were retrospective and conducted in the United States.

Only one study evaluated body mass index (BMI) as a potential risk factor. In this prospective Taiwanese study, a higher BMI, evaluated as a continuous variable, was inversely associated with ESPT development (OR 0.77; 95% CI 0.66-0.90; *P* = 0.001).^25^

Pyriform sinus involvement was described exclusively in the study by Kim et al.,^22^ with a positive association (OR 9.23; 95% CI 3.40-25.08; *P* < 0.00001). The study had a prospective design and was carried out in Taiwan.

Finally, both betel quid chewing and a family history of cancer were not significantly associated with a higher risk of ESPTs in HNSCC patients.^25^^,^^28^^,^^29^^,^^31^^,^^35^ Three studies described betel quid chewing (two prospective and one retrospective studies) and two studies described family history of cancer (one prospective and one retrospective studies), all reporting *P*-values >0.08. All studies were conducted in Asian countries.

#### Risk factors for metachronous ESPTs

Ten studies specifically evaluated metachronous ESPTs and were therefore summarized descriptively.

Male sex was reported as a risk factor by Yamato et al.,^16^ although this was not confirmed by Milliet et al.^33^ Both studies had comparable retrospective sample sizes, but the study by Milliet et al.^33^ differed by the multicenter design of the trial.

Several studies identified primary tumor location as an important determinant of risk. Patients with oropharyngeal and hypopharyngeal tumors had a significantly higher risk compared with those with tongue carcinoma.^16^ Similar results were observed when comparing the oropharynx with the oral cavity.^15^ Tumors of the soft palate were also associated with a higher risk compared with those of the tongue base.^33^ Collectively, these findings support oropharyngeal location as a consistent risk factor.

The presence of LVLs was associated with increased risk.^17^^,^^21^ The presence of dysplasia within these lesions was also identified as a risk factor.^17^^,^^31^

Tobacco exposure demonstrated a significant association, particularly among patients with more than 10 pack-years.^33^ Alcohol consumption was associated with an increased risk in the retrospective study by Milliet et al.^33^ (*n* = 1291), but this finding was not replicated by the prospective study by Chen et al.^31^ (*n* = 175).

Negative p16 status and postoperative radiotherapy following the index tumor were also reported as risk factors.^33^

## Discussion

This systematic literature review consolidates the current evidence on clinical risk factors for ESPTs in patients with HNSCC. Meta-analyses identified significant associations for male sex, advanced tumor stage, alcohol consumption, and primary tumors located in the oropharynx or hypopharynx, whereas smoking did not show a significant pooled effect despite frequent investigation.

The elevated risk associated with oropharyngeal and hypopharyngeal tumors and alcohol consumption may be explained by the principle of *field cancerization*, which reflects the carcinogenic impact of shared mucosal exposure to alcohol and other agents across the upper aerodigestive tract. The higher risk observed in males may be partially attributed to higher alcohol consumption rates among men, reflecting increased exposure to an important etiologic factor.^36^, ^37^, ^38^, ^39^ It could be hypothesized that alcohol consumption thereby acts as a potential confounding factor for male sex. However, due to the design of the present study, additional statistical analyses were not feasible. A multivariate analysis could provide further insight into this relationship and should be considered in future research.

Advanced tumor stage was also noticed as a risk factor for ESPT. However, patients with advanced disease generally have poor prognosis, limiting potential benefit from early detection of ESPT via screening.^40^, ^41^, ^42^ Delaying esophageal endoscopy for screening purposes until ∼1 year after HNSCC diagnosis allows targeting patients with better prognosis and response to therapy while avoiding unnecessary procedures in those with limited life expectancy. The non-significant results regarding smoking habits are unexpected. Five studies were included, of which only two studies showed statistically significant results.^22^^,^^23^^,^^26^^,^^30^^,^^31^ Five studies were excluded due to their lack of a clear distinction between current, former, and non-smokers in their definitions of smoking status. Their alternative classifications (e.g. >10 cigarettes/week for ≥6 months) were incompatible with this framework. Regarding sample size, the study of Chen et al.^31^ included a relatively small cohort (*n* = 175), whereas the other four studies enrolled more than 300 patients each. All studies were conducted in Asian countries. The absence of smoking as a significant risk factor across the included studies is likely multifactorial. Firstly, it may partly be explained by the competing risk of mortality, as smokers are more likely to die from other smoking-related diseases before developing or being diagnosed with an ESPT.^43^ In addition, heterogeneity in study populations may dilute the independent effect of smoking. Variability in exposure assessment, such as the use of crude categorizations (e.g. ever versus never smoking) in several cohort studies, may further contribute to misclassification and attenuation of risk estimates. Finally, in the studies by Lim et al.^23^ and Kim et al.,^22^ a high baseline prevalence of smoking and limited follow-up may reduce exposure contrast and statistical power, further obscuring potential associations. Collectively, these factors may explain the lack of a consistent association and should be considered when interpreting the findings.

Older age has been suggested as a protective factor against the development of ESPTs, although this finding appears controversial.^19^^,^^22^^,^^23^^,^^29^^,^^32^^,^^35^ A plausible explanation is the influence of survivorship bias: younger patients with HNSCC generally have longer post-treatment life expectancy, providing a greater time window for the accumulation of genetic mutations and exposure to carcinogenic risk factors. Consequently, their cumulative lifetime risk for developing ESPTs may be higher compared with older patients. Additionally, age-related biological differences might play a role. Younger patients often have different tumor biology, possibly reflecting different carcinogenic pathways and lifestyle behaviors.^44^ Conversely, older patients may experience a decline in metabolic activity or changes in mucosal regenerative capacity that could reduce the likelihood of new tumor initiation.^45^

BMI was evaluated in only one study, which showed a significant inverse association with the development of ESPTs.^20^ This might be explained by the fact that a low BMI is often associated with extensive alcohol consumption, which is a major etiologic risk factor for both HNSCC and esophageal SCC (conform our meta-analysis).^46^ Moreover, in several cancers, including HNSCC, there is an observed ‘obesity paradox’, in which overweight or mildly obese patients have better outcomes and lower rates of recurrence or SPTs. The mechanism remains unclear, but may relate to higher metabolic reserve, lower likelihood of severe cachexia or sarcopenia, and possibly differential patterns of treatment tolerance or comorbidity interactions.^47^

Negative p16 status and prior radiotherapy have also been proposed as possible risk factors, though the current evidence is limited and warrants further validation.^18^^,^^25^^,^^33^^,^^34^ An important consideration regarding the studies assessing p16 status is that the study by Milliet et al.,^33^ which reported a significant association between negative p16 status and ESPT, included only patients with oropharyngeal HNSCC, whereas Wang et al.^25^ also included patients with hypopharyngeal HNSCC.

Betel quid chewing and family history of cancer showed no significant association with ESPT risk.^25^^,^^28^^,^^29^^,^^31^^,^^35^ The absence of a significant association may reflect limited statistical power, regional variability in exposure, and confounding. These factors might still be associated with HNSCC, but their role in esophageal carcinogenesis remains unclear from the literature used in this systematic review.

Our study has several limitations. Firstly, comparing different studies and combining them in a meta-analysis was challenging due to the heterogeneity across studies. For example, different definitions were used for the same risk factors, particularly for smoking and alcohol consumption. In addition, studies evaluating hypopharyngeal tumors often reported imprecise effect estimates with wide CIs, likely reflecting limited sample sizes for this subgroup. Secondly, we combined studies describing risk factors for both synchronous and metachronous ESPTs assuming that these risk factors are the same for both time points. Restricting the analysis to metachronous tumors alone yielded only 10 studies, with substantial heterogeneity precluding meta-analysis. Therefore, the inclusion criteria were expanded to encompass studies evaluating synchronous tumors. Thirdly, it is of importance that most of these studies describe Asian populations. Results from these studies do not automatically apply to non-Asian populations as there are epidemiological differences between these regions.^48^^,^^49^ We therefore strongly encourage further research in non-Asian countries.

In summary, the risk of developing an ESPT in patients with HNSCC appears to vary according to several factors, including male sex, advanced tumor stage, primary tumor location in the oropharynx or hypopharynx, and excessive alcohol consumption. Our systematic review provides novel insights by focusing on a specific and clinically relevant population. To our knowledge, most existing reviews report on the overall incidence of SPTs in patients with head and neck cancer, without assessing risk factors for ESPTs specifically.^5^^,^^50^ In addition, reviews that do evaluate risk factors typically pool all SPT locations, thereby overlooking site-specific differences.^51^ By focusing exclusively on ESPTs, our study allows for a more precise assessment of relevant risk factors. Our findings suggest that certain patient subgroups may be at relatively higher risk for ESPT development. However, based on the current evidence, the identification of a clearly defined high-risk population suitable for targeted screening remains premature. Further research is needed to establish and validate comprehensive prediction models that can more accurately estimate an individual’s risk of ESPT. Such models could eventually serve as the foundation for developing individualized, risk-based surveillance or screening strategies. Our current study may serve as a guide for identifying factors that should be considered for inclusion in future models. Although some authors have proposed the implementation of periodic esophageal endoscopic screening, particularly in high-risk groups, no formal recommendations currently exist, and the optimal timing and frequency of such screening remain uncertain.^9^^,^^52^^,^^53^ Future large-scale, prospective, and methodologically standardized studies are required to refine risk stratification, validate predictive factors, and determine whether targeted endoscopic surveillance can improve clinical outcomes in HNSCC survivors.

## Declaration of AI and Ai-Assisted Technologies in the WRITING PROCESS

During the preparation of this work the authors used ChatGPT for data collection through a predefined prompt. ChatGPT was also used to adjust and perfect the English vocabulary in this paper. After using this tool/service, the authors reviewed and edited the content as needed and take full responsibility for the content of the published article.

## References

[1] Bray F., Laversanne M., Sung H. (2024). Global cancer statistics 2022: GLOBOCAN estimates of incidence and mortality worldwide for 36 cancers in 185 countries. CA Cancer J Clin.

[2] Bertolini F., Trudu L., Banchelli F. (2021). Second primary tumors in head and neck cancer patients: the importance of a “tailored” surveillance. Oral Dis.

[3] Morris L.G.T., Sikora A.G., Patel S.G., Hayes R.B., Ganly I. (2011). Second primary cancers after an index head and neck cancer: subsite-specific trends in the era of human papillomavirus-associated oropharyngeal cancer. J Clin Oncol.

[4] Rennemo E., Zätterström U., Boysen M. (2008). Impact of second primary tumors on survival in head and neck cancer: an analysis of 2,063 cases. Laryngoscope.

[5] Maan A.D.I., van de Ven S.E.M., Keereweer S., Cornelissen R., Siersema P.D., Koch A.D. (2025). Screening for second primary tumors in the aerodigestive tract in non-Asian populations with head and neck cancer – systematic review and meta-analysis. ESMO Gastrointest Oncol.

[6] Brands M.T., Campschroer G., Merkx M.A.W., Verbeek A.L.M., van Dijk B.A.C., Geurts S.M.E. (2021). Second primary tumours after squamous cell carcinoma of the oral cavity. Eur J Surg Oncol.

[7] Overwater A., Rueb K., Elias S.G., de Bree R., Weusten B.L.A.M. (2022). Esophageal second primary tumors in patients with head and neck squamous cell carcinoma: incidence, risk factors, and overall survival. Am J Gastroenterol.

[8] van Tilburg L., van de Ven S.E.M., de Jonge P.J.F. (2023). Endoscopic screening of the upper gastrointestinal tract for second primary tumors in patients with head and neck cancer in a Western country. Endoscopy.

[9] Brands M.T., Merkx M.A.W., Geurts S.M.E., Brennan P.A. (2017). Should we be rethinking follow-up for oral cancer patients?. J Oral Pathol Med.

[10] Brands M.T., Brennan P.A., Verbeek A.L.M., Merkx M.A.W., Geurts S.M.E. (2018). Follow-up after curative treatment for oral squamous cell carcinoma. A critical appraisal of the guidelines and a review of the literature. Eur J Surg Oncol.

[11] Richtlijnen Database. n.d. https://richtlijnendatabase.nl/richtlijn/hoofd-halstumoren_sept_2023/startpagina_-_hoofd-halstumoren_2023.html.

[12] De Bock T., Maan A., De Cock D., Verhoeven R., Arjun K., Marieke B. (2025). Risk factors associated with second primary esophageal cancer in patients with head and neck squamous cell carcinoma. http://osf.io/hg2zr.

[13] Ouzzani M., Hammady H., Fedorowicz Z., Elmagarmid A. (2016). Rayyan-a web and mobile app for systematic reviews. Syst Rev.

[14] Slim K., Nini E., Forestier D., Kwiatkowski F., Panis Y., Chipponi J. (2003). Methodological index for non-randomized studies (MINORS): development and validation of a new instrument. ANZ J Surg.

[15] Petit T., Georges C., Jung G.M. (2001). Systematic esophageal endoscopy screening in patients previously treated for head and neck squamous-cell carcinoma. Ann Oncol.

[16] Yamamoto E., Shibuya H., Yoshimura R.I., Miura M. (2002). Site specific dependency of second primary cancer in early stage head and neck squamous cell carcinoma. Cancer.

[17] Muto M., Hironaka S., Nakane M., Boku N., Ohtsu A., Yoshida S. (2002). Association of multiple Lugol-voiding lesions with synchronous and metachronous esophageal squamous cell carcinoma in patients with head and neck cancer. Gastrointest Endosc.

[18] Hashibe M., Ritz B., Le A.D., Li G., Sankaranarayanan R., Zhang Z.F. (2005). Radiotherapy for oral cancer as a risk factor for second primary cancers. Cancer Lett.

[19] Chow T.L., Lee D.T.Y., Choi C.Y., Chan T.T.F., Lam S.H. (2009). Prediction of simultaneous esophageal lesions in head and neck squamous cell carcinoma: a multivariate analysis. Arch Otolaryngol Head Neck Surg.

[20] Wang W.L., Lee C.T., Lee Y.C. (2011). Risk factors for developing synchronous esophageal neoplasia in patients with head and neck cancer. Head Neck.

[21] Hori K., Okada H., Kawahara Y. (2011). Lugol-voiding lesions are an important risk factor for a second primary squamous cell carcinoma in patients with esophageal cancer or head and neck cancer. Am J Gastroenterol.

[22] Kim D.H., Gong E.J., Jung H.Y. (2014). Clinical significance of intensive endoscopic screening for synchronous esophageal neoplasm in patients with head and neck squamous cell carcinoma. Scand J Gastroenterol.

[23] Lim H., Kim D.H., Jung H.Y. (2015). Clinical significance of early detection of esophageal cancer in patients with head and neck cancer. Gut Liver.

[24] Huang Y.C., Lee Y.C., Tseng P.H. (2016). Regular screening of esophageal cancer for 248 newly diagnosed hypopharyngeal squamous cell carcinoma by unsedated transnasal esophagogastroduodenoscopy. Oral Oncol.

[25] Wang W.L., Wang Y.C., Chang C.Y. (2016). Human papillomavirus infection on initiating synchronous esophageal neoplasia in patients with head and neck cancer. Laryngoscope.

[26] Gong E.J., Kim D.H., Ahn J.Y. (2016). Routine endoscopic screening for synchronous esophageal neoplasm in patients with head and neck squamous cell carcinoma: a prospective study. Dis Esophagus.

[27] Wang Y.K., Chuang Y.S., Wu T.S. (2017). Endoscopic screening for synchronous esophageal neoplasia among patients with incident head and neck cancer: prevalence, risk factors, and outcomes. Int J Cancer.

[28] Ni X.G., Zhang Q.Q., Zhu J.Q., Wang G.Q. (2018). Hypopharyngeal cancer associated with synchronous oesophageal cancer: risk factors and benefits of image-enhanced endoscopic screening. J Laryngol Otol.

[29] Wang Y.K., Chen W.C., Lai Y.H. (2019). Influence of tea consumption on the development of second esophageal neoplasm in patients with head and neck cancer. Cancers (Basel).

[30] Ho S.Y., Tsang R.K.Y. (2020). Value of oesophagoscopy and bronchoscopy in diagnosis of synchronous malignancies in patients with head and neck squamous cell carcinomas. BMC Cancer.

[31] Chen Y.H., Wang Y.K., Chuang Y.S. (2020). Endoscopic surveillance for metachronous esophageal squamous cell neoplasms among head and neck cancer patients. Cancers (Basel).

[32] Thakur K., Singh C.A., Thakar A. (2021). Prevalence of synchronous ESCN in head and neck cancer: a single-institution perspective. Laryngoscope.

[33] Milliet F., Bozec A., Schiappa R. (2021). Metachronous second primary neoplasia in oropharyngeal cancer patients: impact of tumor HPV status. A GETTEC multicentric study. Oral Oncol.

[34] Zhu X., Zhou J., Zhou L., Zhang M., Gao C., Tao L. (2022). Association between postoperative radiotherapy for young-onset head and neck cancer and long-term risk of second primary malignancy: a population-based study. J Transl Med.

[35] Wang C.C., Hsu M.H., Lee C.T. (2024). Prognostic significances of systemic inflammatory response markers in patients with synchronous esophageal and head and neck cancers. Head Neck.

[36] Pujol A., Llansana A., Pérez-Ugarte L., Sauter B., Quer M., León X. (2023). Second esophageal neoplasms after head and neck index tumor: incidence, risk factors and prognosis. Acta Otorrinolaringol Esp (Engl Ed).

[37] León X., Del Prado Venegas M., Orús C., Kolañczak K., García J., Quer M. (2005). Metachronous second primary tumours in the aerodigestive tract in patients with early stage head and neck squamous cell carcinomas. Eur Arch Otorhinolaryngol.

[38] Slaughter D.P., Southwick H.W., Smejkal W. (1953). Field cancerization in oral stratified squamous epithelium; clinical implications of multicentric origin. Cancer.

[39] World Health Organization (2024). Alcohol. https://www.who.int/news-room/fact-sheets/detail/alcohol.

[40] Kamran S.C., Qureshi M.M., Jalisi S., Salama A., Grillone G., Truong M.T. (2018). Primary surgery versus primary radiation-based treatment for locally advanced oropharyngeal cancer. Laryngoscope.

[41] Tsai Y.T., Chen W.C., Chien C.Y. (2020). Treatment patterns and survival outcomes of advanced hypopharyngeal squamous cell carcinoma. World J Surg Oncol.

[42] Abrahão R., Perdomo S., Pinto L.F.R. (2020). Predictors of survival after head and neck squamous cell carcinoma in South America: the InterCHANGE Study. JCO Glob Oncol.

[43] Rojewski A.M., Baldassarri S., Cooperman N.A. (2016). Exploring issues of comorbid conditions in people who smoke. Nicotine Tob Res.

[44] Toner M., O’Regan E.M. (2009). Head and neck squamous cell carcinoma in the young: a spectrum or a distinct group? Part 1. Head Neck Pathol.

[45] Zheng H., Zhang C., Wang Q., Feng S., Fang Y., Zhang S. (2022). The impact of aging on intestinal mucosal immune function and clinical applications. Front Immunol.

[46] Choi Y.J., Lee D.H., Han K.D. (2017). Joint effects of low body mass index and alcohol consumption on developing esophageal squamous cell cancer: a Korean Nationwide Population-Based Cohort Study. Asian Pac J Cancer Prev.

[47] Fattouh M., Chang G.Y., Ow T.J. (2019). Association between pretreatment obesity, sarcopenia, and survival in patients with head and neck cancer. Head Neck.

[48] Zhang J., Jiang Y., Wu C. (2015). Comparison of clinicopathologic features and survival between eastern and western population with esophageal squamous cell carcinoma. J Thorac Dis.

[49] Zhang H.Z., Jin G.F., Shen H.B. (2012). Epidemiologic differences in esophageal cancer between Asian and Western populations. Chin J Cancer.

[50] Coca-Pelaz A., Rodrigo J.P., Suárez C. (2020). The risk of second primary tumors in head and neck cancer: a systematic review. Head Neck.

[51] Salcedo-Bellido I., Requena P., Mateos R. (2022). Factors associated with the development of second primary tumours in head and neck cancer patients. Eur J Cancer Care (Engl).

[52] Chung C.S., Liao L.J., Wu C.Y. (2022). Endoscopic screening for second primary tumors of the esophagus among head and neck cancer patients. Front Oncol.

[53] Ye Y.C., Wang Y.P., Chang T.E. (2024). Routine image-enhanced endoscopic surveillance for metachronous esophageal squamous cell neoplasms in head and neck cancer patients. Esophagus.

