# Supplementary material for: Risk factors for second primary esophageal cancer after head and neck squamous cell carcinoma: a systematic review and meta-analysis
Source: ESMO Gastrointest Oncol. 2026 Jun 30;13(Pt B):100351. doi: 10.1016/j.esmogo.2026.100351 (PMC13459574; doi:10.1016/j.esmogo.2026.100351)
Supplement: Supplementary file 1 [file mmc1.docx]

**Supplementary data:**

**Form 1: Search strategy PubMed**

1. Research question

What are the risk factors for developing a second primary oesophageal cancer in patients with head and neck squamous cell carcinoma?

2. PICO format

| P: problem/population | Patients with head and neck squamous cell carcinoma (HNSCC) |
| --- | --- |
| I: intervention/exposure | Risk factors |
| C: comparison | / |
| O: outcome | Development of a second primary oesophageal cancer |

3. Search strategy PubMed

#1 Head and neck squamous cell carcinoma

"Squamous Cell Carcinoma of Head and Neck"[Mesh] OR "HNSCC"[tiab] OR "Head And Neck Squamous Cell Carcinoma*"[tiab] OR "Carcinoma, Squamous Cell of Head and Neck"[tiab] OR "Squamous Cell Carcinoma of the Head and Neck"[tiab] OR "Squamous Cell Carcinoma, Head And Neck"[tiab] OR "Oral Squamous Cell Carcinoma*"[tiab] OR "Squamous Cell Carcinoma of the Mouth"[tiab] OR "Oral Cavity Squamous Cell Carcinoma"[tiab] OR "Squamous Cell Carcinoma of the Larynx"[tiab] OR "Laryngeal Squamous Cell Carcinoma"[tiab] OR "Squamous Cell Carcinoma of the Nasal Cavity"[tiab] OR "Oropharyngeal Squamous Cell Carcinoma"[tiab] OR "Hypopharyngeal Squamous Cell Carcinoma"[tiab] OR "Oral Tongue Squamous Cell Carcinoma"[tiab]

#2 Risk factors

"Risk Factors"[Mesh] OR "causality"[Mesh] OR "Factor, Risk"[tiab] OR "Risk Factor*"[tiab] OR "Population* at Risk"[tiab] OR "Risk Score*"[tiab] OR "Score, Risk"[tiab] OR "Risk Factor Score*"[tiab] OR "Score, Risk Factor"[tiab] OR "Health Correlate*"[tiab] OR "Correlates, Health"[tiab] OR "Alcohol Consumption"[tiab] OR "Smoking"[tiab] OR "Tobacco Use"[tiab] OR "Betel Quid"[tiab] OR "HPV Infection"[tiab] OR "Human Papillomavirus"[tiab] OR "Gastroesophageal Reflux"[tiab] OR "GERD"[tiab] OR "Genetic Predisposition"[tiab] OR "Radiotherapy"[tiab] OR "Radiation Exposure"[tiab] "Causalit*"[tiab] OR "Enabling Factor*"[tiab] OR "Factor*, Enabling"[tiab] OR "Multifactorial Causalit*"[tiab] OR "Multiple Causation*"[tiab] OR "Predisposing Factor*"[tiab] OR "Factor*, Predisposing"[tiab] OR "Reinforcing Factor*"[tiab] OR "Factor*, Reinforcing"[tiab] OR "Factors, Reinforcing"[tiab] OR "Causation*"[tiab] OR "etiology"[Subheading] OR etiology[Text Word]

#3 Cancer, second primary

"Neoplasms, Second Primary"[Mesh] OR “esophageal neoplasms”[Mesh] OR "Neoplasm, Second Primary"[tiab] OR "Second Primary Neoplasm*"[tiab] OR "Second Malignanc*"[tiab] OR "Malignanc*, Second"[tiab] OR "Second Neoplasm*"[tiab] OR "Neoplasm*, Second"[tiab] OR "Metachronous Second Primary Neoplasm*"[tiab] OR "Metachronous Neoplasm*"[tiab] OR "Neoplasm*, Therapy Related"[tiab] OR "Neoplasm*, Therapy-Related"[tiab] OR "Therapy-Associated Neoplasm*"[tiab] OR "Therapy Related Neoplasm*"[tiab] OR "Treatment-Associated Neoplasm*"[tiab] OR "Treatment Related Neoplasm*"[tiab] OR "Cancer*, Second Primary"[tiab] OR "Second Primary Cancer*"[tiab] OR "Second Cancer*"[tiab] OR "Therapy-Associated Cancer*"[tiab] OR "Treatment-Associated Cancer*"[tiab] OR "Therapy-Related Cancer*"[tiab] OR "Treatment-Related Cancer*"[tiab] OR "Esophageal Neoplasm*"[tiab] OR "Neoplasm*, Esophageal"[tiab] OR "Esophagus Neoplasm*"[tiab] OR "Neoplasm*, Esophagus"[tiab] OR "Esophageal Cancer*"[tiab] OR "Cancer*, Esophageal"[tiab] OR "Cancer of the Esophagus"[tiab] OR "Esophagus Cancer*"[tiab] OR "Cancer*, Esophagus"[tiab]

#4

#1 AND #2 AND #3

#5

"Animals"[Mesh] NOT "Humans"[Mesh]

#1 AND #2 AND #3 NOT #5

**Form 2: Search strategy Embase**

(‘head and neck squamous cell carcinoma’/exp OR 'HNSCC':ti,ab,kw OR 'Head And Neck Squamous Cell Carcinoma*':ti,ab,kw OR 'Carcinoma, Squamous Cell of Head and Neck':ti,ab,kw OR 'Squamous Cell Carcinoma of the Head and Neck':ti,ab,kw OR 'Squamous Cell Carcinoma, Head And Neck':ti,ab,kw OR 'Oral Squamous Cell Carcinoma*':ti,ab,kw OR 'Squamous Cell Carcinoma of the Mouth':ti,ab,kw OR 'Oral Cavity Squamous Cell Carcinoma':ti,ab,kw OR 'Squamous Cell Carcinoma of the Larynx':ti,ab,kw OR 'Laryngeal Squamous Cell Carcinoma':ti,ab,kw OR 'Squamous Cell Carcinoma of the Nasal Cavity':ti,ab,kw OR 'Oropharyngeal Squamous Cell Carcinoma':ti,ab,kw OR 'Hypopharyngeal Squamous Cell Carcinoma':ti,ab,kw OR 'Oral Tongue Squamous Cell Carcinoma':ti,ab,kw )

AND

(‘risk’/exp OR ‘causality’/exp OR 'Factor, Risk':ti,ab,kw OR 'Risk Factor*':ti,ab,kw OR 'Population* at Risk':ti,ab,kw OR 'Risk Score*':ti,ab,kw OR 'Score, Risk':ti,ab,kw OR 'Risk Factor Score*':ti,ab,kw OR 'Score, Risk Factor':ti,ab,kw OR 'Health Correlate*':ti,ab,kw OR 'Correlates, Health':ti,ab,kw OR 'Alcohol Consumption':ti,ab,kw OR 'Smoking':ti,ab,kw OR 'Tobacco Use':ti,ab,kw OR 'Betel Quid':ti,ab,kw OR 'HPV Infection':ti,ab,kw OR 'Human Papillomavirus':ti,ab,kw OR 'Gastroesophageal Reflux':ti,ab,kw OR 'GERD':ti,ab,kw OR 'Genetic Predisposition':ti,ab,kw OR 'Radiotherapy':ti,ab,kw OR 'Radiation Exposure':ti,ab,kw OR 'Causalit*':ti,ab,kw OR 'Enabling Factor*':ti,ab,kw OR 'Factor*, Enabling':ti,ab,kw OR 'Multifactorial Causalit*':ti,ab,kw OR 'Multiple Causation*':ti,ab,kw OR 'Predisposing Factor*':ti,ab,kw OR 'Factor*, Predisposing':ti,ab,kw OR 'Reinforcing Factor*':ti,ab,kw OR 'Factor*, Reinforcing':ti,ab,kw OR 'Causation*':ti,ab,kw)

AND

(‘second primary neoplasm’/exp OR ‘esophagus tumor’/exp OR 'esophageal neoplasms':ti,ab,kw OR 'Neoplasm, Second Primary':ti,ab,kw OR 'Second Primary Neoplasm*':ti,ab,kw OR 'Second Malignanc*':ti,ab,kw OR 'Malignanc*, Second':ti,ab,kw OR 'Second Neoplasm*':ti,ab,kw OR 'Neoplasm*, Second':ti,ab,kw OR 'Metachronous Second Primary Neoplasm*':ti,ab,kw OR 'Metachronous Neoplasm*':ti,ab,kw OR 'Neoplasm*, Therapy Related':ti,ab,kw OR 'Neoplasm*, Therapy-Related':ti,ab,kw OR 'Therapy-Associated Neoplasm*':ti,ab,kw OR 'Therapy Related Neoplasm*':ti,ab,kw OR 'Treatment-Associated Neoplasm*':ti,ab,kw OR 'Treatment Related Neoplasm*':ti,ab,kw OR 'Cancer*, Second Primary':ti,ab,kw OR 'Second Primary Cancer*':ti,ab,kw OR 'Second Cancer*':ti,ab,kw OR 'Therapy-Associated Cancer*':ti,ab,kw OR 'Treatment-Associated Cancer*':ti,ab,kw OR 'Therapy-Related Cancer*':ti,ab,kw OR 'Treatment-Related Cancer*':ti,ab,kw OR 'Esophageal Neoplasm*':ti,ab,kw OR 'Neoplasm*, Esophageal':ti,ab,kw OR 'Esophagus Neoplasm*':ti,ab,kw OR 'Neoplasm*, Esophagus':ti,ab,kw OR 'Esophageal Cancer*':ti,ab,kw OR 'Cancer*, Esophageal':ti,ab,kw OR 'Cancer of the Esophagus':ti,ab,kw OR 'Esophagus Cancer*':ti,ab,kw OR 'Cancer*, Esophagus':ti,ab,kw)

**Form 3: Search strategy Web of Science**

(TS=("Squamous Cell Carcinoma of Head and Neck" OR "HNSCC" OR "Head And Neck Squamous Cell Carcinoma*" OR "Carcinoma, Squamous Cell of Head and Neck" OR "Squamous Cell Carcinoma, Head And Neck" OR "Oral Squamous Cell Carcinoma*" OR "Squamous Cell Carcinoma of the Mouth" OR "Oral Cavity Squamous Cell Carcinoma" OR "Squamous Cell Carcinoma of the Larynx" OR "Laryngeal Squamous Cell Carcinoma" OR "Squamous Cell Carcinoma of the Nasal Cavity" OR "Oropharyngeal Squamous Cell Carcinoma" OR "Hypopharyngeal Squamous Cell Carcinoma" OR "Oral Tongue Squamous Cell Carcinoma")

AND

(TS=("Factor, Risk" OR "Causalit*" OR "Risk Factor*" OR "Population* at Risk" OR "Risk Score*" OR "Score, Risk" OR "Risk Factor Score*" OR "Score, Risk Factor" OR "Health Correlates*" OR "Correlates, Health" OR "Alcohol Consumption" OR "Smoking" OR "Tobacco Use" OR "Betel Quid" OR "HPV Infection" OR "Human Papillomavirus" OR "Gastroesophageal Reflux" OR "GERD" OR "Genetic Predisposition" OR "Radiotherapy" OR "Radiation Exposure" OR "Enabling Factor*" OR "Factor*, Enabling" OR "Multifactorial Causalit*" OR "Multiple Causation*" OR "Predisposing Factor*" OR "Factor*, Predisposing" OR "Reinforcing Factor*" OR "Factor*, Reinforcing" OR "Causation*")

AND

(TS=("Neoplasm, Second Primary" OR "Second Primary Neoplasm*" OR "Second Malignanc*" OR "Malignanc*, Second" OR "Second Neoplasm*" OR "Neoplasm*, Second" OR "Metachronous Second Primary Neoplasm*" OR "Metachronous Neoplasm*" OR "Neoplasm*, Therapy Related" OR "Therapy-Associated Neoplasm*" OR "Therapy Related Neoplasm*" OR "Treatment-Associated Neoplasm*" OR "Treatment Related Neoplasm*" OR "Cancer*, Second Primary" OR "Second Primary Cancer*" OR "Second Cancer*" OR "Therapy-Associated Cancer*" OR "Treatment-Associated Cancer*" OR "Therapy-Related Cancer*" OR "Treatment-Related Cancer*" OR "Esophageal Neoplasm*" OR "Neoplasm*, Esophageal" OR "Esophagus Neoplasm*" OR "Neoplasm*, Esophagus" OR "Esophageal Cancer*" OR "Cancer*, Esophageal" OR "Cancers*, Esophageal" OR "Cancer of the Esophagus" OR "Esophagus Cancer*" OR "Cancer*, Esophagus")

**Form 4: Prompt for data extraction using ChatGPT**

Instruction for PDF Data Extraction:

Please analyze each PDF I upload and extract the following data for each study. Provide the output in CSV format, using a semicolon (;) as the delimiter for easy pasting into Excel. Use the following headers in this exact order:

*General Study Information:*

- Study ID (Optional – leave blank or use a short identifier if needed)
- First Author
- Title
- Year of Publication
- Journal
- Country
- Study Design
- Sample Size

*Tumor-Related Clinical Data:*

- Identified Risk Factors for Esophageal Second Primary Tumor
  - List of risk factors evaluated in the study.
- Definition of Each Risk Factor
  - How each risk factor is defined or operationalized in the study (e.g., smoking = >10 pack-years, alcohol use = ≥3 drinks/day).
- Prevalence of Each Risk Factor
  - Proportion or number of patients in the study with each risk factor.
- Association Between Each Risk Factor and Esophageal Second Primary Tumor
  - Reported strength of association (e.g., hazard ratio, odds ratio, correlation coefficient, or qualitative description).

*Head and Neck Tumor Details:*

- Head and Neck Tumor - Location (e.g., larynx, pharynx, oral cavity)
- Head and Neck Tumor - TNM Classification
- Head and Neck Tumor - Histology
- Head and Neck Tumor - Treatment

*Esophageal Second Primary Tumor Details:*

- Esophageal Tumor - Location
- Esophageal Tumor - Incidence
- Esophageal Tumor - Histology
- Time Until Second Primary Esophageal Tumor (from primary diagnosis)
- Mortality Due to Second Primary Esophageal Tumor

*Screening Protocol:*

Screening Protocol Used for Patients with Head and Neck Tumors (e.g., endoscopy, frequency, timing)

*MINORS Criteria – Non-Comparative Items (All Studies):*

- MINORS - A Stated Aim of the Study
- MINORS - Inclusion of Consecutive Patients
- MINORS - Prospective Collection of Data
- MINORS - Endpoint Appropriate to the Study Aim
- MINORS - Unbiased Evaluation of Endpoints
- MINORS - Follow-Up Period Appropriate to the Major Endpoint
- MINORS - Loss to Follow-Up Not Exceeding 5%

*MINORS Criteria – Comparative Studies Only (Leave blank if the study is not comparative):*

- MINORS - Control Group Receiving Gold Standard Intervention
- MINORS - Use of Contemporary Groups
- MINORS - Baseline Equivalence of Groups
- MINORS - Prospective Calculation of Sample Size
- MINORS - Statistical Analyses Adapted to Study Design

**Addendum:**

*Supplementary Table 1: Overview of the included articles and quality grading according to the MINORS criteria [14]. Quality of non-comparative studies was defined as low (score ≤10), medium (score 11–14), and high (score ≥15). Quality of comparative studies was defined as low (score ≤15), medium (score 16–21), and high (score ≥22).*

| **Authors** | **Title** | **Quality** |
| --- | --- | --- |
| Petit T. et al. | Systematic esophageal endoscopy screening in patients previously treated for head and neck squamous-cell carcinoma | Medium |
| Yamamoto E. et al. | Site Specific Dependency of Second Primary Cancer in Early Stage Head and Neck Squamous Cell Carcinoma | Medium |
| Muto M. et al. | Association of multiple Lugol-voiding lesions with synchronous and metachronous esophageal squamous cell carcinoma in patients with head and neck cancer | Medium |
| Hashibe M. et al. | Radiotherapy for oral cancer as a risk factor for second primary cancers | Medium |
| Chow T.L. et al. | Prediction of Simultaneous Esophageal Lesions in Head and Neck Squamous Cell Carcinoma | Medium |
| Wang W.L. et al. | Human papillomavirus infection on initiating synchronous esophageal neoplasia in patients with head and neck cancer | Medium |
| Hori K. et al. | Lugol-Voiding Lesions Are an Important Risk Factor for a Second Primary Squamous Cell Carcinoma in Patients With Esophageal Cancer or Head and Neck Cancer | Medium |
| Kim D.H. et al. | Clinical significance of intensive endoscopic screening for synchronous esophageal neoplasm in patients with head and neck squamous cell carcinoma | Medium |
| Lim H. et al. | Clinical significance of early detection of esophageal cancer in patients with head and neck cancer | Medium |
| Huang Y.C. et al. | Regular screening of esophageal cancer for 248 newly diagnosed hypopharyngeal squamous cell carcinoma by unsedated transnasal esophagogastroduodenoscopy | Medium |
| Wang W.L. et al. | Risk factors for developing synchronous esophageal neoplasia in patients with head and neck cancer | Medium |
| Gong E.J. et al. | Routine endoscopic screening for synchronous esophageal neoplasm in patients with head and neck squamous cell carcinoma: a prospective study | Medium |
| Wang Y.K. et al. | Endoscopic screening for synchronous esophageal neoplasia among patients with head and neck squamous cell carcinoma | Medium |
| Ni X.G. et al. | Hypopharyngeal cancer associated with synchronous oesophageal cancer: risk factors and benefits of image-enhanced endoscopic screening | Medium |
| Wang Y.K. et al. | Influence of tea consumption on development of esophageal second primary tumor in head and neck cancer patients | Medium |
| Ho S.Y. et al. | Value of oesophagoscopy and bronchoscopy in diagnosis of synchronous malignancies in patients with head and neck squamous cell carcinomas | Medium |
| Chen Y.H. et al. | Endoscopic surveillance for metachronous esophageal squamous cell neoplasms among head and neck cancer patients | Medium |
| Thakur K. et al. | Prevalence of Synchronous ESCN in Head and Neck Cancer: A Single‐Institution Perspective | Medium |
| Brands M.T. et al. | Time patterns of recurrence and second primary tumors in a large cohort of patients treated for oral cavity cancer | Medium |
| Milliet F. et al. | Metachronous second primary neoplasia in oropharyngeal cancer patients: Impact of tumor HPV status | Medium |
| Zhu X. et al. | Association between postoperative radiotherapy for young-onset head and neck cancer and long-term risk of second primary malignancy | Medium |
| Wang C.C. et al. | Prognostic significances of systemic inflammatory response markers in patients with synchronous esophageal and head and neck cancers | Medium |
